# Effect of chromatin modifiers on the plasticity and immunogenicity of small-cell lung cancer

**DOI:** 10.1038/s12276-022-00905-x

**Published:** 2022-12-12

**Authors:** Nicole A. Kirk, Kee-Beom Kim, Kwon-Sik Park

**Affiliations:** 1 Department of Microbiology, Immunology, and Cancer Biology, School of Medicine, University of Virginia, Charlottesville, VA 22908 USA; 2 BK21 FOUR KNU Creative BioResearch Group, School of Life Sciences, Kyungpook National University, Daegu, 41566 Republic of Korea

**Keywords:** Cancer, Diseases

## Abstract

Tumor suppressor genes (TSGs) are often involved in maintaining homeostasis. Loss of tumor suppressor functions causes cellular plasticity that drives numerous types of cancer, including small-cell lung cancer (SCLC), an aggressive type of lung cancer. SCLC is largely driven by numerous loss-of-function mutations in TSGs, often in those encoding chromatin modifiers. These mutations present a therapeutic challenge because they are not directly actionable. Alternatively, understanding the resulting molecular changes may provide insight into tumor intervention strategies. We hypothesize that despite the heterogeneous genomic landscape in SCLC, the impacts of mutations in patient tumors are related to a few important pathways causing malignancy. Specifically, alterations in chromatin modifiers result in transcriptional dysregulation, driving mutant cells toward a highly plastic state that renders them immune evasive and highly metastatic. This review will highlight studies in which imbalance of chromatin modifiers with opposing functions led to loss of immune recognition markers, effectively masking tumor cells from the immune system. This review also discusses the role of chromatin modifiers in maintaining neuroendocrine characteristics and the role of aberrant transcriptional control in promoting epithelial-to-mesenchymal transition during tumor development and progression. While these pathways are thought to be disparate, we highlight that the pathways often share molecular drivers and mediators. Understanding the relationships among frequently altered chromatin modifiers will provide valuable insights into the molecular mechanisms of SCLC development and progression and therefore may reveal preventive and therapeutic vulnerabilities of SCLC and other cancers with similar mutations.

## Small-cell lung cancer: cellular plasticity and immune evasion tricks

Investigating how mutated protein complexes trigger immune evasion during small-cell lung cancer (SCLC) could yield possible therapeutic approaches for this intractable disease. SCLC is driven by loss-of-function mutations in tumour suppressor genes, which in turn alter the activity of chromatin modifiers, protein complexes that alter chromatin, the mixture of DNA and proteins forming chromosomes. Kwon-Sik Park at the University of Virginia in Charlottesville, USA, and co-workers have reviewed current understanding of the mechanisms involved. Studies show that imbalances in different chromatin modifier groups can result in the loss of tumour recognition markers, effectively hiding tumour cells from the immune system. The hyperactivity of one specific chromatin modifier regulates cancer cell plasticity, shifting cells from epithelial to a less specialised type, meaning they can differentiate and spread through the body more quickly.

## Introduction

Lung cancer is the leading cause of cancer-related deaths worldwide. The two main types of lung cancer are small-cell lung cancer (SCLC) and non-small-cell lung cancer (NSCLC); the latter is further divided into lung adenocarcinoma, squamous-cell carcinoma, and large-cell carcinoma. These lung cancer types are typically defined by unique immunohistological features and are clinically managed very differently. SCLC accounts for 10 to 15% of all lung cancers^1–3^ and is an exceptionally lethal type of lung cancer with a 5-year survival rate of 7%, which is significantly worse than that of NSCLC (26%)^4,5^. The high mortality of SCLC is mainly due to its highly proliferative and metastatic nature and its rapid development of resistance to genotoxic chemo/radiation therapy. Unlike lung adenocarcinoma, which is frequently driven by druggable alterations such as activating mutations in EGFR, ALK, and KRAS^6,7^, SCLC is driven mostly by unactionable, loss-of-function mutations in genes encoding tumor suppressors, including RB1 and P53^8^.

Recent sequencing studies of the SCLC genome have uncovered a plethora of mutations that may hold the keys to molecular changes within patient tumor cells^6,9^. The most striking feature of the SCLC genome is the fact that both the *TP53* and *RB1* genes are mutated in over 90% of patients^8,9^. Studies of genetically engineered mouse models have shown that concomitant loss of *Rb1* and *Trp53* is required for SCLC initiation but have also suggested roles of additional drivers in progression to full malignancy. A group of chromatin modifiers mutated in 50% of SCLC patient tumors have emerged as putative tumor suppressors given the global impact of their inactivation and their implication in other cancer types^8,10^. They include lysine-specific histone acetyltransferases (e.g., CREBBP and EP300), lysine-specific histone methyltransferases (e.g., KMT2 family proteins, also known as MLLs), lysine-specific histone demethylases (e.g., LSD1 and KDM6A) and components of the SWI/SNF (SWItch/Sucrose Non-Fermentable) chromatin remodeling complex (e.g., SMARCA4/2 and ARID1A/B). The small numbers of patient tumors examined in the studies of the SCLC genome preclude a robust analysis of mutual exclusivity and cooccurrence among the mutations to determine functional relationships; however, the known functional interactions among the proteins on target chromatin led to speculation that the epigenetic dysregulation resulting from the mutations is related to key pathways causing malignancy.

Little is known of the specific dysregulated pathways, except for findings from studies of cell cycle dysregulation in cancer cells-of-origin^11–13^ and studies of preneoplasia progressing to full-blown SCLC. More importantly, much less is known about the impact of mutations in mutant epithelial cells on the stroma of patient tumors. SCLC patients rarely undergo surgical resection of primary tumors because of the high occurrence of metastasis^14–16^. The limited access to patient tumors makes it difficult to gain direct insights into the SCLC tumor microenvironment (TME). To reveal interactions within the TME, synthesis of data from patient tumors and various mouse models is needed to recapitulate the interactions between tumor cells and cellular components of the microenvironment.

Here, we review recent studies suggesting that the balanced actions among various chromatin modifiers are a critical gatekeeper of SCLC cell identity and homeostatic interactions with TME. Alterations in chromatin modifiers largely cause epigenetic dysregulation, leading to transcriptional silencing of immune markers on the surface of mutant cells and effectively leading to immune exclusion within the TME. Concurrently, transcriptional reprogramming enhances cellular plasticity, contributing to the transition between SCLC subtypes and the acquisition of mesenchymal characteristics.

## Role of epigenetic alterations in reshaping the immune landscape in SCLC

Recently, interest has arisen around the potential use of utilizing one’s own immune system to target SCLC through immunotherapies, including immune checkpoint blockade (ICB)^17^. ICB employs antibodies to block immune checkpoint ligands (e.g., PD-L1, CTLA4) to activate cytotoxic T cells (CD8-positive), which induce lysis and killing of target cells^18,19^. Theoretically, SCLC seems to be a perfect candidate for ICB: the high mutational burden of SCLC should present sufficient neoantigens for activating the adaptive immune system, rendering these cells susceptible to ICB-driven immunotherapy^20^. Disappointingly, however, ICB treatment is often ineffective in treating SCLC^21,22^. This is largely due to the “immune cold” nature of SCLC tumors, which are characterized by significantly decreased immune cell infiltration. In an early study assessing levels of immune infiltration within patient samples, low-grade tumors showed higher levels of infiltrating CD8+T cells and macrophages than their high-grade counterparts^23^. Tumors derived from a mouse model of SCLC exhibited alarmingly low levels of immune infiltration, whereas KRAS- and EGFR-driven NSCLC tended to have robust levels of immune infiltration^24^. Furthermore, the immune cells that do penetrate SCLC tumors tend to contribute to an immunosuppressive environment; for example, there are increased proportions of regulatory T cells (Tregs), M2-polarized macrophages, and myeloid-derived suppressor cells (MDSCs)^25^. These cells suppress the activation of other immune effectors by releasing anti-inflammatory cytokines, allowing for the unchecked proliferation of tumor cells. The molecular changes within SCLC cells that alter the immune microenvironment have been of significant interest yet remain poorly understood. Particularly, a role of epigenetic alterations in reshaping the immune microenvironment has recently begun to emerge.

An increasing body of evidence suggests that chromatin modifiers influence how SCLC cells interact with both the adaptive and innate immune systems. Although ubiquitously expressed, the expression of MHC-I (major histocompatibility complex-I), the primary molecule for engaging cytotoxic T cells, is mostly decreased in SCLC tumors^26–28^. Recent studies have implicated polycomb repressive complex 2 (PRC2) in suppressing MHC-I expression and presentation. Deletion of either embryonic ectoderm development (EED) or enhancer of Zeste homolog 2 (EZH2), the core components of PRC2, was sufficient to restore MHC-I expression and cell surface display^29^. EED is involved in recruitment of the other subunits to form PRC2, and EZH2 is a catalytic subunit with histone methyltransferase functions. EZH2-mediated trimethylation of lysine 27 on histone H3 (H3K27me3) in gene promoters and enhancers stimulates DNA compaction. This modification of the histone results in a condensed chromatin structure and thus renders the nearby genes inaccessible to transcriptional machinery, leading to gene repression or silencing^30^. These transcriptional alterations result in insufficient MHC-I display on the cell surface. Furthermore, this activity is often co-opted by many cancers with silencing of various tumor suppressor genes, among which are the genes encoding cell cycle regulators and antigen processing and presentation pathway components. PRC2 was also found to silence genes encoding components of the antigen presentation pathway (APP), including *TAP1/2*, which are responsible for antigenic peptide transportation to the endoplasmic reticulum (ER) and loading onto assembled MHC-I^29,31,32^. Without proper antigen presentation, tumor-associated antigens can no longer be recognized by and subsequently activate T cells. Epigenetic suppression of MHC-I and APP components prevents SCLC cells from being recognized by T cells, particularly cytotoxic T cells, which are the key effectors of immune checkpoint blockade (ICB) therapy. However, it is important to note that losing the expression of this “self-recognition” receptor should render cells susceptible to being targeted by the cellular effectors of the innate immune system, including macrophages, natural killer (NK) cells, and neutrophils. How tumor cells escape this new challenge remains poorly understood.

Macrophages were found to be largely absent in the immune microenvironment of higher grade SCLC tumors, and those that were present were positive for CD163, indicative of an M2 polarized state of cells^23,33,34^. Since such polarization results in loss of the antitumor activity of macrophages, the mechanisms underlying the depletion or inactivation of macrophages remain an important research topic. A recent study suggested a role for CD47 on SCLC cells in evasion of macrophages^33^. CD47 is a cell surface protein on tumor cells that binds to SIRPα (signal regulatory protein alpha) on macrophages, and the protein-protein interaction activates the cytoplasmic domain of SIRPα to trigger intracellular signals leading to inhibition of phagocytic activity^35,36^. Elevated levels of CD47 provide a clear example of transcriptional dysregulation in SCLC contributing to immune evasion, but the mechanisms underlying upregulation of this immune marker in SCLC remain unknown. Beyond the expression of CD47, macrophage activation and chemotaxis to tumors is also mediated by chemokines and cytokines^37^. SCLC tumors with low levels of macrophage infiltration have been found to have low levels of CC chemokine ligand 2 (CCL2) expression, which is a chemokine responsible for the recruitment of monocytes that differentiate into macrophages. Similar to MHC-I, CCL2 was found to be epigenetically silenced by histone methylation at its enhancer mediated by EZH2^38^. In the same study, CCL2 expression was also repressed by DNMT1 methylation of CpG islands at the CCL2 enhancer. Implementation of EZH2 or DNMT1 inhibitors was found to rescue CCL2 expression in SCLC cells. Furthermore, the use of these inhibitors led to increased macrophage recruitment in vivo implantation experiments.

NK cells are another potent effector of the innate immune system. They recognize natural killer group 2, member D ligand (NKG2DL) (including the subsets MICA/B and ULBP) on host cells^39^. Upon recognition and activation, NK cells release perforin and granzymes to induce cytolysis of target cells. Compared to NSCLC cells, SCLC cells have significantly lower levels of NKG2DL^40^. The levels of NKG2DL are negatively correlated with the levels of MYC, a marker of the NEUROD1 subtype of SCLC. MYC works in concert with histone deacetylase 3 (HDAC3) to deacetylate the promoter of NKG2DL (specifically MICA/B) at histone 3 lysine 9 (H3K9Ac), thus removing the transcriptional activation signal. NKG2DL is also recognized by cytotoxic T cells, providing yet another example of epigenetic silencing within SCLC cells that hides them from various components of the immune system. The relationship between NKG2DL and MYC is an example of how the heterogeneity underlying each SCLC subtype contributes to varying levels of immune infiltration within patient tumors.

**Fig. 1:**
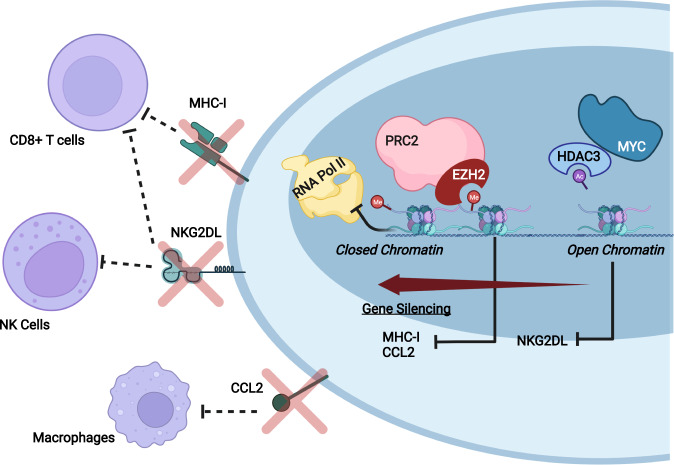
Epigenetic regulation of immune recognition markers in SCLC. The schematic depicts epigenetic control of immunogenicity in SCLC. Hyperactivity of PRC2, particularly increased methylation (H3K27me3) by its catalytic subunit EZH2, leads to transcriptional silencing of MHC-I and CCL2. Loss of these markers masks mutant cells from CD8+T cells and macrophages. HDAC3 removes the transcriptional activation signal (H3K9Ac) on histone tails at the promoter of NKG2DL, leading to DNA compaction. Loss of NKG2DL expression inhibits recognition by NK cells and CD8+T cells. The hyperactivity of these chromatin modifiers in SCLC contributes to a closed chromatin conformation at the regulatory regions of the genome surrounding these immune markers. As a result, these regions become inaccessible to transcriptional machinery.

The ability to circumvent detection by immune surveillance is one of the hallmarks of cancer development^41^, allowing the excessive and sustained proliferation of mutant cells, and is often acquired through aberrant transcriptional regulation of key immune recognition markers (Fig. 1). Epigenetic silencing of MHC-I, CCL2, and NKG2DL allows SCLC cells to avoid recognition and destruction by both the innate and adaptive arms of the immune system.

## Exploiting epigenetic vulnerability for immune therapies

The significant impact of dysregulated chromatin modifiers on immune evasion and depletion in the TME led to the concept that reversing these transcriptional reprogramming events may be a valuable treatment strategy to be employed in combination with existing ICB. EZH2 has been shown to induce widespread changes in the expression of immune markers in SCLC cells, for example, silencing MHC-I and CCL2, thus inhibiting interactions with T cells and macrophages. Use of an EZH2 inhibitor led to a reversal of MHC-I silencing in an SCLC cell line^29^. Similarly, when EZH2 was chemically inhibited in an SCLC cell line, CCL2 expression and monocyte recruitment were restored^38^. The combination of BET and HDAC6 inhibitors showed promising results in various xenograft and allograft models of SCLC mediated by NK cells^42^. The use of a pan-HDAC inhibitor led to a derepression of MICA expression in SCLC cells that express high levels of ASCL1, a classical marker of neuroendocrine (NE) characteristics in SCLC. Furthermore, the use of the pan-HDAC inhibitor trichostatin A (TSA) was also sufficient to increase NK-cell recruitment and activation in allograft experiments^43^. Together, these studies highlight that the use of inhibitors of chromatin modifiers not only changes the expression of several key immune regulatory markers on the surface of SCLC tumor cells but also leads to increased levels of immune infiltration. The combination of an EZH2 inhibitor with a PD-L1 blocker was suggested after examination of an SCLC cohort in which most patients displayed a stark lack of PD-L1 expression while harboring higher levels of EZH2^44^. When combined with existing ICB strategies, targeting these epigenetic regulators may prime the cells for increased recognition by both the adaptive and innate arms of the immune system. While these aberrant epigenetic modifications in SCLC may present interesting targets for therapeutic intervention, it is important to recognize some of the major caveats of epigenetic therapy. These include a lack of specificity both in delivery and in targeting. An early study evaluating the effects of 5-aza-CdR, an inhibitor of DNA methylation, and TSA, an inhibitor of histone deacetylation, laid out an important framework for testing the effects of epigenetic targeted drugs: comparing the results of parallel genetic manipulation of targeted chromatin modifiers to chemically induced outcomes. In the study, the researchers compared the effects of these nonspecific inhibitors with the effects of targeted knockdown of DNMT1 and DNMT3^45^. The study showed the importance of using genetic targeting strategies, such as CRISPR technology, as a means to assess the impact of directly inhibiting a target gene versus the off-target effects of a nonspecific inhibitor.

## Epigenetic control and immunogenicity of SCLC subtypes

SCLC has long been treated as a singular disease. This is reflected in the near universal use of the combination of cytotoxic chemotherapy and radiation to treat SCLC patients. This strategy has proven to be mostly ineffective at halting tumor progression. However, there has been a significant shift in the field with the increasing appreciation of the heterogeneity between and within patient tumors. A molecular subtyping strategy that classifies SCLC cells into three groups (SCLC-A, N, and P) based on the expression of neural lineage-defining transcription factors (ASCL1, NEUROD1, or POU2F3) has recently been proposed^46^. Alternatively, these subtypes can be further subdivided into neuroendocrine (SCLC-A/-N) and nonneuroendocrine (SCLC-P) subtypes. SCLC arises from neuroendocrine cells in the central airway^12^. The neuroendocrine characteristics are often lost during SCLC progression (i.e., there is a transition from neuroendocrine (NE) to non-NE characteristics). Previously, a fourth subtype, SCLC-Y, was proposed based on YAP1 expression, but the relevance of this subtype has since been questioned, and SCLC-Y is currently regarded as a non-NE subtype rather than an independent subtype. Instead, another subtype called the “inflamed” subtype (SCLC-I) has been added to the list, which is characterized by a gene signature with elevated levels of immune checkpoint markers coinciding with increased levels of immune infiltration^47^. Levels of immune infiltration differ by subtype, which will be described in further detail below. Recent emphasis has been placed on discovering what drives the progression of each subtype and the overlapping transformation mechanisms that all subtypes use. Epigenetic control of gene expression culminates in alterations made to the chromatin landscape impacting gene expression. The following sections will describe how aberrant chromatin modifiers drive changes to the chromatin layout within transforming SCLC cells to promote tumor development.

One of the factors deciding SCLC subtype is lysine demethylase 1 (LSD1), which acts as a transcriptional repressor by removing activating monomethylation or dimethylation of histone H3 lysine 4 (H3K4me1/me2) at regions flanking transcription start sites^48–50^. Methylation of H3K4 is a well-established marker of actively transcribed genes^51–54^ Removal of these posttranslational modifications inhibits recruitment and interactions with chromatin remodelers and transcription factors. LSD1 expression was found to be higher in the majority of SCLC samples (at an astounding rate of 98%) compared to adjacent normal tissues, and the protein activity was found to be concentrated at neuronal differentiation genes^55^. Treatment of SCLC cells with an LSD1 inhibitor revealed that sensitivity to this drug could be predicted by the expression levels of members of the MYC family of transcription factors, including MYCL, MYCN, and MYC. Consistent with the patterns of *MYCL*/*MYCN* amplification in the SCLC-A subtype and *MYC* amplification in the SCLC-N subtype^8,46^, the expression of ASCL1 and NEUROD1 in SCLC cell lines representing the SCLC-A and SCLC-N subtypes was dependent on LSD1 activity^56^. Furthermore, LSD1 inhibition impacted the expression of SYP and CHGA, additional NE markers. Blocking LSD1 activity not only decreased the expression of NE markers but also subsequently resulted in activation of the NOTCH signaling pathway, which indicates transcriptional reprogramming toward a non-NE phenotype^50^.

ASCL1 is a key transcription factor in the maintenance of NE features of SCLC cells and is highly expressed in 70% of SCLC cases^46^. ASCL1 expression is often sustained by chromatin modifiers. Similar to LSD1, when the histone demethylase KDM5A was targeted by CRISPR/Cas9 in various SCLC cell lines, the expression of ASCL1 and the NE markers CHGA and SYP decreased^57^. These SCLC cell lines often grow as clusters in suspension; however, after targeting KDM5A, the cells became adherent in culture. This transition in culture is often a sign of SCLC cells losing their NE characteristics. Furthermore, KDM5A was found to bind to the NOTCH2 promoter and to several NOTCH target genes, silencing their expression and silencing the overall NOTCH signaling pathway. LSD1 and KDM5A are examples of proteins that epigenetically control cellular identity by sustaining the NE features of SCLC cells.

The previous section described the relative lack of immune cells within the SCLC microenvironment. Further examination of the correlation between immune infiltration within patient tumors and circulating levels of immune cells revealed that immune cell infiltration within the tumor varies based on the degree of NE characteristics of the tumor^25,58^. NE-high tumors (SCLC-A/-N) were found to have decreased levels of T-cell and NK-cell infiltration compared to NE-low tumors (SCLC-P). These low levels of immune infiltration were in contrast with the increased levels of T cells, NK cells, and macrophages within NE-low tumors. These tumors represent the emerging inflamed subtype (SCLC-I)^47^. Genetically engineered mouse models (GEMMs) of the two NE-high subtypes of SCLC (RP: *Rb1*^lox/lox^*;Trp53*^lox/lox^ and RPR2: *Rb1*^lox/lox^*;Trp53*^lox/lox^*;Rbl2*^lox/lox^)^11,13^ recapitulate what is seen in patient samples. The tumors from the RP and RPR2 GEMMs, both of which are SCLC-A, displayed higher levels of immune markers than the RPM GEMM (*Rb1*^lox/lox^*;Trp53*^lox/lox^*; H11*^lox-stop-lox-MycT58A^) tumors, which are SCLC-N. The levels of immune infiltration within these tumors also followed the patterns seen in patient tumors, with RPM tumors displaying the lowest amount of T-cell infiltration^58^. The analysis of patient data and validation in GEMMs confirms the classification of SCLC as being immune cold, with the NEUROD1 subset of tumors showing the greatest lack of infiltrating immune cells.

Not only did NEUROD1 tumors have the lowest levels of infiltrating immune cells, but the T cells present were also found to be composed of higher levels of regulatory T cells (Tregs) and cytotoxic T cells (CD8+) displaying an exhausted phenotype than the respective levels in SCLC-A tumors^25^, contributing to an immunosuppressive environment. These results highlight the heterogeneity of immune infiltration composition based on SCLC subtype: NE-high subtypes (SCLC-A/SCLC-N) are primarily immune-suppressive based on immune cell infiltration levels and surface levels of immune recognition markers. While these NE characteristics have been shown to be sustained through the efforts of various chromatin modifiers, including LSD1 and KDM5A, how epigenetic regulation of NE features contributes to the varying expression levels of immune markers remains to be determined.

NE-high tumors consistently show high levels of immune exclusion due to low expression levels of MHC-I and TAP1, both of which are epigenetically silenced by PRC2^29^. Interestingly, SCLC patient samples with low levels of NE markers (ASCL1-low, CGRP-low) tended to have high levels of MHC-I expression that correlated with the expression of EMT markers^59^. In the same study, the researchers discovered a subset of cells in the H69 SCLC cell line that were enriched for low NE expression and an increase in TAP1 and MHC-I expression. Rescue of the expression of these markers was correlated with an increase in levels of H3K27Ac, a transcriptional activation marker opposing the repressive markers of PRC2. This suggests that as SCLC cells transition from an NE to a non-NE phenotype, they undergo transcriptional reprogramming, which impacts not only lineage plasticity but also immunogenicity. This notion was further exemplified by a recent study assessing the effects of LSD1 inhibition in various SCLC cell lines^60^. LSD1 inhibition led to a loss of NE features that coincided with the rescue of the expression of MHC-I and other components of the APP pathway. LSD1-mediated restoration of MHC-I and APP component expression sensitized RPR2 tumors to PD-L1 blockade, resulting in increased infiltration of cytotoxic T cells. This study provides a clear example of the efficacy of targeting chromatin modifiers to treat SCLC by enhancing the response to ICB. It also provides a novel link between the simultaneous regulation of NE characteristics and immune recognition markers in SCLC.

## Epigenetic regulation of cell plasticity during SCLC development and progression

At the time of diagnosis, two-thirds of SCLC tumors have already metastasized^61,62^. This alarmingly high rate of metastasis presents a therapeutic challenge. It has been suggested that the metastatic switch in SCLC is driven by “chromatin reprogramming”^63^. This idea was supported by an analysis of patient samples and tumors derived from RP GEMMs in which the expression of the neural transcription factor NFIB was increased in metastases^64–66^. Although the mechanism remains undiscovered, the elevated levels of NFIB correlated with a hyperaccessible chromatin state. Interestingly, in melanoma, NFIB was found to increase EZH2 expression^67^. As described in the previous section, EZH2 is a potent regulator of epigenetic dynamics in SCLC. Chromatin modifiers control transcription by fostering either an open or closed conformation. Closed chromatin, often resulting from amplified levels of histone methylation, is marked by an increase in DNA compaction around histones and thus renders these regions of the genome inaccessible to transcriptional machinery. In contrast, acetylation of histones along with nucleosome sliding or even eviction increases accessibility. Anomalous control of these accessibility states is a key mechanism by which chromatin modifiers drive cellular plasticity.

There appears to be a pattern emerging: the shifts in chromatin organization during tumor progression impact the transcription of key genes; regions of the genome containing the promoters of crucial oncogenes tend to be maintained in a hypomethylated (accessible) state, while regions that include the promoters of tumor suppressor genes tend to be hypermethylated (inaccessible)^68,69^. However, the mechanism behind these selective methylation patterns remains unknown. In the previous section, this pattern was shown to manifest in the loss of cellular identity, including the loss of NE characteristics and immune markers. This portion of the review will discuss how imbalance of chromatin modifiers propels the transformation from an epithelial to a mesenchymal state. Epithelial-mesenchymal transition (EMT) is a major cellular mechanism underlying metastasis and is driven by a number of genetic and phenotypic changes. These changes are mainly related to cell-cell interactions resulting in increased mobility, allowing tumor cells to migrate and invade secondary regions throughout the body. The most common EMT- associated changes include loss of expression of the cell adhesion molecule E-cadherin (CDH1) and upregulation of the expression of N-cadherin (CDH2) and vimentin^70^. Furthermore, these transcriptional changes are maintained through increased expression of the following EMT-associated transcription factors: SNAIL, SLUG, TWIST, ZEB1, and ZEB2^70^. Just as the transition between SCLC subtypes is being increasingly appreciated as a gradual process in which transitioning cells often display overlap of subtype markers, EMT is no longer regarded as a binary event and has begun to be defined as a progressive transformation^70^. In accordance with the guidelines from ‘the EMT International Association’ (TEMTIA), the ability of epithelial cells to undergo this transformation will be referred to as “epithelial-mesenchymal plasticity” (EMP) for the remainder of this review.

While metastasis is often associated with the later stages of cancer progression, the tendency for SCLC to exhibit early metastasis has raised the question of whether these cells contain an inherent propensity to metastasize^71^. This pattern of early metastases is recapitulated in experimental models of SCLC, including GEMMs, which often feature very few initiating mutations: the RP model has only conditional loss of *Rb1* and *Trp53*, the RPR2 model has additional deletion of *Rbl2* (also known as *p130*), and the RPM model additionally features a hyperactive form of MYC^11,13^. In particular, cells isolated from the primary tumors of these mice initially grow as spheroid aggregates in suspension, thus indicating that these primary tumor cells alter cell-matrix and cell-cell interactions and display a cellular phenotype conducive to invasion. While the paucity of mutations may support the notion of an inherent capacity of mouse SCLC to metastasize, the nearly intact cell-matrix and cell-cell interactions seen in *Rb1*/*Trp53*-deficient preneoplastic cells (hereafter called preSCs), derived from early-stage lesions in GEMMs, imply the need for additional drivers to induce drastic cellular transformation^72,73^. A recent review laid out the mechanisms of metastasis in SCLC^74^; however, this section will focus on how epigenetic dysregulation, particularly that induced through the actions of chromatin modifiers, drives EMP in SCLC through epigenetic silencing of epithelial markers and a subsequent increase in mesenchymal characteristics.

## Role of EZH2 in epithelial-mesenchymal plasticity of SCLC

SCLC patients often have a history of smoking. Chronic cigarette smoke exposure (CSE) has been linked to inducing mutations involving C:G > A:T transversions^8^. Prolonged CSE of human bronchial epithelial cells (HBECs) has also been found to induce aberrant transcriptional control, promoting the transition to a mesenchymal phenotype^75–77^. These smoke-exposed HBECs acquire a mesenchymal phenotype, as indicated by elongation. At the molecular level, these cells lack CDH1 expression while simultaneously gaining expression of vimentin and CDH2, both of which are mesenchymal markers. The expression levels of several lncRNAs, including HOTAIR and MALAT1, are increased in HBECs after smoke exposure^78^. Just as chromatin-modifying complexes control transcription by altering DNA-histone interactions, lncRNAs can regulate transcription through recruitment and assembly of various transcription factors and chromatin-modifying complexes^79^. Further exploration of the effects of continuous smoke exposure on HBECs revealed that the transcriptional reprogramming was driven by increased EZH2 activity mediated by the lncRNA metastasis-associated in lung adenocarcinoma transcript 1 (MALAT1)^80^. CSE was found to be positively correlated with MALAT1 expression, which was found to be necessary for maintaining EZH2 expression. Previous studies have provided a link between prolonged smoke exposure and acquiring a mesenchymal phenotype. Furthermore, these studies showed a correlative relationship between CSE and increased EZH2 expression and raise an interesting question: can chronic CSE-induced epigenetic dysregulation prime untransformed NE cells for EMT occurring after the acquisition of additional oncogenic alterations?

In many cancers, such as NSCLC, oncogenic alterations involve the upregulation of potent drivers, such as KRAS and EGFR^6,7^. However, in SCLC, the alterations tend to involve the gradual accumulation of loss-of-function mutations. EZH2 hyperactivity has been proven to have widespread effects on transcriptional reprogramming in SCLC. Its apparent hyperactivity may be attributed to frequent loss-of-function mutations in the chromatin-modifying complexes that antagonize EZH2 and compete for occupancy at regulatory regions of the genome. Chromatin modifiers form interconnected, complex networks that often work in concert with or in opposition to each other. PRC2 and methyltransferases are often associated with gene silencing, particularly through methylation of lysine residues 9 and 27 on histone 3 (H3K9/27). In contrast, histone acetyltransferases, such as CREBBP/EP300 (also known as CBP/p300), work together with various complexes, such as the SWI/SNF complex, to promote active transcription by relaxing DNA around histones and increasing accessibility to transcriptional machinery. CREBBP was found to be a potent tumor suppressor in SCLC^73^. Loss of CREBBP-mediated histone acetylation results in transcriptional reprogramming within *Rb1*/*Trp53*-deficient preSCs and promotes tumorigenic transformation of these preneoplastic cells. The actions of CREBBP and PRC2 often converge at the regulatory regions of the same genes. EZH2 silences CDH1 expression by methylating histones at its promoter (resulting in H3K27me3)^78,81^. In preSCs, targeting CREBBP is sufficient to reduce the expression of CDH1 and tight junction proteins and concomitantly increase the expression of mesenchymal markers, including vimentin and ZEB1. The decrease in CDH1 expression caused by the loss of CREBBP parallels the reduction in CDH1 caused by EZH2-mediated promoter methylation. These findings support the idea that EZH2 hyperactivity in SCLC is crucial to the loss of competitors for chromatin occupancy. Given the high frequency of chromatin modifiers harboring mutations in SCLC tumors, there is a need to investigate how interactions between these complexes drive tumor development and progression through transcriptional reprogramming.

Emerging evidence has begun to show a strong correlation between a mesenchymal phenotype and chemoresistance. PARP1 has been shown to be highly upregulated in SCLC^82^, thus providing a potential vulnerability that can be targeted therapeutically. Increased expression of both CDH1 and SLFN11, a helicase involved in the response to DNA damage, was found to be a reliable predictor of sensitivity to PARP inhibitors, suggesting a negative association between EMT and drug sensitivity^83^. Although that study provided evidence of actionable predictors of therapeutic sensitivity in SCLC, it also reflects a commonality in treating SCLC, in which patient tumors show an initial yet fleeting response to treatment. SLFN11 expression was found to be frequently repressed in cell lines derived from patients who have undergone some form of chemotherapy^84^. Furthermore, the expression of SLFN11 was found to be directly regulated by an all-encompassing methylation pattern of the histones surrounding and inclusive of the SLFN11 gene body mediated by EZH2. In a set of SLFN11-low, previously treated SCLC cell lines, SLFN11 expression could be rescued through the administration of EZH2 inhibitors. Supporting the notions of a strong mesenchymal signature and chemoresistance, SLFN11 expression was found to be negatively correlated with the expression of TWIST1. This is yet another example of the effects of EZH2 hyperactivity in promoting the pervasiveness of SCLC through both acquired resistance and EMP.

## Expanding landscape of epigenetic dysregulation underlying cell plasticity

While numerous studies have highlighted the importance of EZH2 hyperactivity in SCLC development, they have only focused on one piece of the puzzle. Methyltransferases, such as EZH2, often become overexpressed in SCLC, making them attractive targets for therapeutic intervention. However, it is interesting to note that thus far, in SCLC patient samples, there are no documented copy number alterations of these proteins leading to their increased expression, suggesting that they are also subject to epigenetic control. Therefore, looking at a patient’s genetic profile will not indicate increased levels of EZH2 activity, but it will provide information about mutations occurring on the other side of the chromatin modification spectrum. There are numerous other chromatin modifiers that have a competitive relationship with PRC2, many of which are frequently mutated in SCLC. However, their role in SCLC progression remains significantly understudied. Therefore, the following section will summarize studies describing how frequently mutated chromatin modifiers contribute to cellular plasticity in the context of other cancers. These studies may provide important starting points to explore in SCLC.

The SWI/SNF chromatin-modifying complex is one of the most commonly mutated complexes across various cancer types^85^, including SCLC. It works in concert with CREBBP to promote an open chromatin conformation through nucleosome sliding at competing sites with PRC2. The SWI/SNF complex is an ATP-dependent chromatin-remodeling complex that is made up of 15 subunits that form various combinations. SMARCA4/2 (BRG1/BRM1) and ARID1A/B (BAF250A/B) are integral subunits of the complex and are recurrently mutated in patient tumors in a mutually exclusive fashion^86^. While individual component mutations are less frequent (<5%) in SCLC, the mutations all result in SWI/SNF dysfunction. Thus, the compounded frequency (~20%) of the mutations provides a strong rationale for investigating the role of the SWI/SNF complex in SCLC. Especially given the antagonistic role of the SWI/SNF complex and PRC2, understanding the impact of SWI/SNF subunits may provide insight into therapeutic vulnerabilities of patient tumors harboring mutations related to EZH2 inhibitor resistance, as seen above, with CBP/P300 mutations increasing vulnerability to these inhibitors. *SMARCA4* is also frequently mutated in NSCLC. Monitoring tumor formation in NSCLC GEMMs of lung adenocarcinoma (KPS: *Kras*^LSL-G12D/+^; *Trp53*^lox/lox^; *Smarca4*^lox/lox^) revealed that regardless of the cell type in which the alleles were targeted (alveolar type II epithelial cells or club cells), *Smarca4* loss led to increased metastasis^87^. In studies of both gastric cancer and pancreatic ductal adenocarcinoma (PDAC), analysis of patient samples and available cell lines revealed that loss of either *Smarca4* or *Arid1a* led to increased metastasis and decreased CDH1 expression^88–90^. Furthermore, *SMARCA4* loss in triple-negative breast cancer (TNBC) cell lines was associated with an increased mesenchymal signature, including increased expression of *SNAIL*, *TWIST*, and *CDH2* and increased HIPPO pathway activity^91^. The HIPPO pathway is associated with an invasive phenotype^92^, and one of its key mediators is YAP1: a key non-NE marker found to be upregulated upon loss of ASCL1 and NEUROD1 in SCLC. In the study of TNBC, loss of *SMARCA4* led to increased YAP signaling activity, and in a specific subtype (SL subtype: MDA-MB-468 cells), it was found to lead to increased expression of both *YAP* and its downstream targets. As previously described, the NE to non-NE transition in SCLC is frequently regulated by the chromatin modifiers LSD1 and KDM5A. The connection between the SCLC subtype and EMP signatures is gaining interest. Transcriptional analysis of cells isolated from RPM GEMMs identified gradual loss of NE marker expression (ASCL1, NEUROD1) that coincided with increased expression of YAP1 and mesenchymal markers (TWIST, SNAIL, ZEB1, ZEB2)^93^. Loss of the expression of these key SWI/SNF complex components may contribute to increased plasticity in SCLC through NE dedifferentiation and the accumulation of mesenchymal markers.

UTX (KDM6A) is a histone demethylase and is also found to be recurrently mutated in SCLC, but the impact of these mutations on SCLC development remains unexplored. UTX knockdown in NSCLC cells leads to a modest decrease in CDH1 expression^94^. Furthermore, in colon cancer, UTX was found to interact with CREBBP at the CDH1 promoter^95^. This interaction complements the inhibition of CDH1 transcription by PRC2 through increased methylation at the CDH1 promoter^78^. Together, these studies highlight the antagonistic relationship between UTX and PRC2. In a study evaluating the metastatic potential of breast cancer stem cells, UTX was found to work in concert with LSD1 and HDAC1 to remove the transcriptional activation signal H3K4me2 at the promoters of EMT-TFs, including SNAIL, ZEB1, and ZEB2, and thus inhibit their expression^96^. Interestingly, LSD1 localization at these promoters was found to be dependent on UTX expression. Given the contrasting levels of NE-high SCLC cells within primary tumors and NE-low cells within metastases, loss of UTX may be one of the molecular switches priming cells for EMT by increasing the expression of EMT-TFs.

**Fig. 2:**
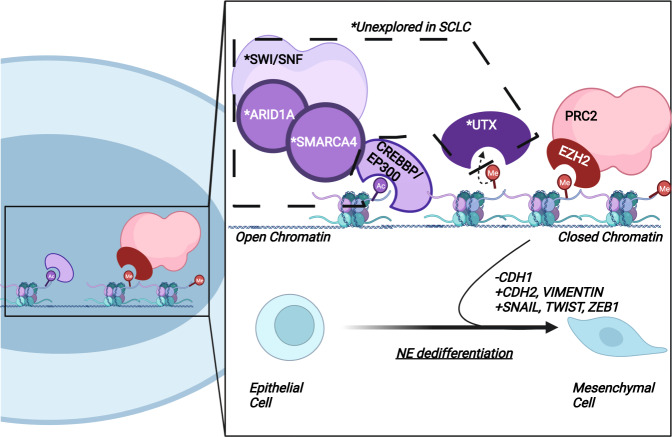
Chromatin modifiers control cellular plasticity in SCLC and other cancers. In this schematic, we depict a scenario in which mutations in chromatin modifiers that promote active transcription (SWI/SNF components, UTX) contribute to EZH2 hyperactivity in SCLC. As shown in both SCLC and other cancers, loss of these components along with EZH2-mediated chromatin remodeling leads to loss of epithelial markers and increased expression of mesenchymal markers and EMT-TFs. These transcriptional changes result in mutant cells acquiring a mesenchymal phenotype, priming these cells for metastasis. *Frequently mutated yet unexplored in SCLC.

Current studies in SCLC suggest that EZH2 hyperactivity kickstarts the transition from an epithelial to a mesenchymal state. While EZH2’s methyltransferase capabilities lead to transcriptional repression of key EMP effectors, it does not act in isolation. Epigenetic regulation by chromatin modifiers is a highly dynamic process. There are many chromatin modifiers that compete with EZH2 for chromatin occupancy, which are frequently mutated in SCLC patients. However, their role in the context of SCLC development remains rarely explored. Based on our review of studies exploring these chromatin modifiers in various cancers and the established relationship between CREBBP mutations and EZH2 in SCLC^73^, we propose that loss-of-function mutations in chromatin modifiers (e.g., *ARID1A*, *SMARCA4*, *UTX*) lead to loss of NE identity and increased plasticity (Fig. 2).

## Conclusions

**Fig. 3:**
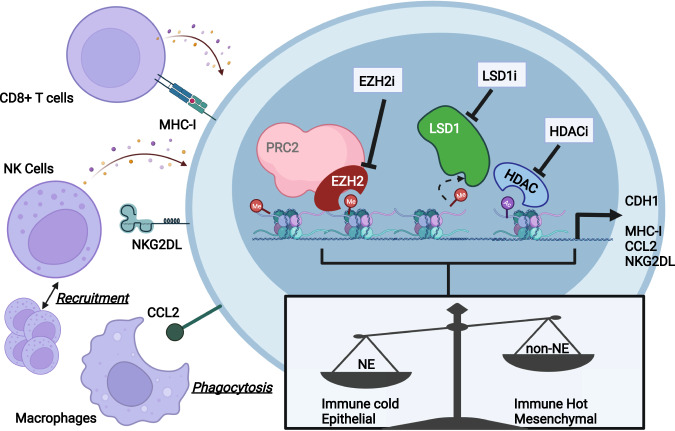
Convergence of aberrant chromatin modifications in SCLC. In this schematic, we emphasize that alterations in chromatin modifiers are related to key processes in SCLC development: immune evasion, loss of cell identity, and epithelial-mesenchymal plasticity. Chromatin modifiers (including EZH2, LSD1, HDAC) can be targeted therapeutically to restore the expression of immune markers (MHC-I, CCL2, NKG2DL) to increase recognition by immune cells and their recruitment to the TME.

In recent years, SCLC research has made remarkable progress in understanding the biology of SCLC (a recalcitrant disease) and is beginning to uncover critical interactions between tumor cells and the immune landscape of the TME through the creation and implementation of many physiologically relevant mouse models. Synthesis of these models along with knowledge gained from patient samples suggests that despite the substantial heterogeneity in the SCLC genome mutational profile, the mutations tend to be related to a few critical pathways that drive tumor development and progression. This review has focused on the emerging role of chromatin modifiers as the driving force behind key pathways promoting tumor development. Within mutant cells, an imbalance among chromatin modifiers (e.g., hyperactivity of methyltransferases and loss-of-function of chromatin remodelers) promotes immune evasion, increased plasticity, and the acquisition of mesenchymal characteristics (Fig. 3). Further examination of the processes underlying these processes suggests that they are not separate events in the progression towards malignancy. For instance, recent studies have demonstrated the importance of tumor cells losing immune recognition markers as they disseminate to secondary locations^97,98^. This is evident in the interactions between NEUROD1-high SCLC and NK cells. SCLC-N has the lowest levels of immune infiltration and the highest numbers of metastases. Maintenance of these NE features is controlled by chromatin modifiers (LSD1, KDM5A)^50,56,57^. NK cells were found to be strictly excluded from mouse SCLC-N tumors^58^. Exploring the role of NK cells in tumor progression revealed that these cytotoxic cells did not have an apparent effect on primary tumor formation, but once activated, they were found to be key mediators controlling the metastatic spread of SCLC. Furthermore, NK cells identify their targets through recognition of NKG2DL. However, in SCLC, NKG2DL is transcriptionally silenced due in part to the cooperation of MYC and HDAC3. These findings highlight the role of chromatin modifiers as gatekeepers of the overlapping processes of immune recognition and metastatic spread in SCLC. Targeting these chromatin modifiers shows promising results in restoring the expression of immune markers and even recruiting immune cells to the SCLC TME (Fig. 3).

Perhaps the most robust example of convergence despite such heterogeneity in SCLC is EZH2-mediated transcriptional dysregulation. EZH2 acts as a master regulator of SCLC plasticity. EZH2 hyperactivity in SCLC results in transcriptional reprogramming crucial for various aspects of both immune evasion and EMP in SCLC. It is responsible for silencing both MHC-I and CCL2, masking tumor cells from both the innate and adaptive arms of the immune system. As a regulator of lineage plasticity, EZH2 regulates the expression of CDH1 and interacts with lncRNAs to control the expression of various mesenchymal markers. EZH2 itself is subject to transcriptional regulation by NFIB. These examples show that EZH2 hyperactivity promotes SCLC tumor formation by affecting both the immunogenicity and plasticity of transforming cells. Extensive studies have compiled lists of EZH2 target genes; however, the mechanisms involved in EZH2 recruitment to target gene sites in the genome remain comparatively underexplored. In SCLC, it was discovered that the transcriptional repressor chromodomain Y-like (CDYL) recruits EZH2 to the cyclin-dependent kinase inhibitor 1 C (CDKN1C) promoter, leading to its silencing and further contributing to chemoresistance^99^. Other mediators of EZH2 localization at other target genes in SCLC remain unknown. It has been proposed that EZH2, and other Polycomb group (PcG) proteins, may be recruited to CpG islands through lncRNAs. In particular, HOTAIR was found to recruit EZH2 to the CDH1 promoter in nasopharyngeal carcinoma (NPC), leading to its transcriptional repression^100^. Given the increased expression of lncRNAs following CSE and the apparent hyperactivity of EZH2 in SCLC, exploring this relationship in the context of EZH2 localization to target genes in transformed SCLC cells may provide further insight into additional molecular vulnerabilities. Additionally, understanding the mechanisms of EZH2 localization may provide broader insight into the aberrant methylation landscape occurring during tumor progression across various cancers, including insight into how oncogenes tend to be selectively spared from hypermethylation to allow their persistent accessibility and expression. While its comprehensive role of EZH2 in SCLC has been appreciated in numerous studies, an understanding of the role of chromatin modifiers that antagonize EZH2 is lacking. These chromatin modifiers have been found to harbor recurrent mutations in SCLC and may represent therapeutic vulnerabilities. For example, loss of SMARCA4 in NSCLC cell lines led to sensitization to EZH2 inhibitors^101^. Understanding which patients may benefit most from therapy targeting epigenetic regulators may enhance the effectiveness of current immunotherapies^44,60^. Exploring the relationship among frequently altered chromatin modifiers will strengthen our understanding of the pathways exploited by transcriptional dysregulation during SCLC progression.

## References

[1] Rodriguez E, Lilenbaum RC (2010). Small Cell Lung Cancer: Past, Present, and Future. Curr. Oncol. Rep..

[2] American Cancer Society. Cancer Facts & Figures 2022. (2022).

[3] Califano R, Abidin AZ, Peck R, Faivre-Finn C, Lorigan P (2012). Management of Small Cell Lung Cancer. Drugs..

[4] Byers LA, Rudin CM (2015). Small cell lung cancer: Where do we go from here?. Cancer.

[5] Rudin CM, Brambilla E, Faivre-Finn C, Sage J (2021). Small-cell lung cancer. Nat. Rev. Dis. Prim..

[6] Friedlaender A, Drilon A, Weiss GJ, Banna GL, Addeo A (2020). KRAS as a druggable target in NSCLC: Rising like a phoenix after decades of development failures. Cancer Treat. Rev..

[7] Jordan EJ (2017). Prospective Comprehensive Molecular Characterization of Lung Adenocarcinomas for Efficient Patient Matching to Approved and Emerging Therapies. Cancer Disco..

[8] George J (2015). Comprehensive genomic profiles of small cell lung cancer. Nature.

[9] Peifer M (2012). Integrative genome analyses identify key somatic driver mutations of small-cell lung cancer. Nat. Genet..

[10] Augert A (2017). Small Cell Lung Cancer Exhibits Frequent Inactivating Mutations in the Histone Methyltransferase KMT2D/MLL2: CALGB 151111 (Alliance). J. Thorac. Oncol..

[11] Meuwissen R (2003). Induction of small cell lung cancer by somatic inactivation of both Trp53 and Rb1 in a conditional mouse model. Cancer Cell..

[12] Park K-S (2011). Characterization of the cell of origin for small cell lung cancer. Cell Cycle..

[13] Schaffer BE (2010). Loss of p130 Accelerates Tumor Development in a Mouse Model for Human Small-Cell Lung Carcinoma. Cancer Res..

[14] van Meerbeeck JP, Fennell DA, De Ruysscher DK (2011). Small-cell lung cancer. Lancet.

[15] Gazdar AF, Bunn PA, Minna JD (2017). Small-cell lung cancer: what we know, what we need to know and the path forward. Nat. Rev. Cancer..

[16] Wang S (2017). Survival changes in patients with small cell lung cancer and disparities between different sexes, socioeconomic statuses and ages. Sci. Rep..

[17] Reck M (2013). Ipilimumab in combination with paclitaxel and carboplatin as first-line therapy in extensive-disease-small-cell lung cancer: results from a randomized, double-blind, multicenter phase 2 trial†. Ann. Oncol..

[18] Topalian SL, Drake CG, Pardoll DM (2015). Immune Checkpoint Blockade: A Common Denominator Approach to Cancer Therapy. Cancer Cell..

[19] Wei SC, Duffy CR, Allison JP (2018). Fundamental Mechanisms of Immune Checkpoint Blockade Therapy. Cancer Disco..

[20] Hellmann MD (2018). Tumor Mutational Burden and Efficacy of Nivolumab Monotherapy and in Combination with Ipilimumab in Small-Cell Lung Cancer. Cancer Cell..

[21] Ishii H (2015). Significance of Programmed Cell Death-Ligand 1 Expression and its Association with Survival in Patients with Small Cell Lung Cancer. J. Thorac. Oncol..

[22] Yamane H (2015). Programmed cell death protein 1 and programmed death-ligand 1 are expressed on the surface of some small-cell lung cancer lines. Am. J. Cancer Res..

[23] Eerola AK, Soini Y, Pääkkö P (2000). A high number of tumor-infiltrating lymphocytes are associated with a small tumor size, low tumor stage, and a favorable prognosis in operated small cell lung carcinoma. Clin. Cancer Res. J. Am. Assoc. Cancer Res..

[24] Busch SE (2016). Lung Cancer Subtypes Generate Unique Immune Responses. J. Immunol..

[25] Chan JM (2021). Signatures of plasticity, metastasis, and immunosuppression in an atlas of human small cell lung cancer. Cancer Cell..

[26] Doyle A (1985). Markedly decreased expression of class I histocompatibility antigens, protein, and mRNA in human small-cell lung cancer. J. Exp. Med..

[27] Funa K, Gazdar AF, Minna JD, Linnoila RI (1986). Paucity of beta 2-microglobulin expression on small cell lung cancer, bronchial carcinoids and certain other neuroendocrine tumors. Lab. Investig. J. Tech. Methods Pathol..

[28] Restifo NP (1993). Identification of human cancers deficient in antigen processing. J. Exp. Med..

[29] Burr ML (2019). An Evolutionarily Conserved Function of Polycomb Silences the MHC Class I Antigen Presentation Pathway and Enables Immune Evasion in Cancer. Cancer Cell..

[30] Schuettengruber B, Chourrout D, Vervoort M, Leblanc B, Cavalli G (2007). Genome Regulation by Polycomb and Trithorax Proteins. Cell.

[31] Kaer LV, Ashton-Rickardt PG, Ploegh HL, Tonegawa S (1992). TAP1 mutant mice are deficient in antigen presentation, surface class I molecules, and CD4−8+T cells. Cell.

[32] Sandberg JK, Chambers BJ, Van Kaer L, Kärre K, Ljunggren H-G (1996). TAP1-deficient mice select a CD8+T cell repertoire that displays both diversity and peptide specificity. Eur. J. Immunol..

[33] Weiskopf K (2016). CD47-blocking immunotherapies stimulate macrophage-mediated destruction of small-cell lung cancer. J. Clin. Invest..

[34] Dora D (2021). Characterization of Tumor-Associated Macrophages and the Immune Microenvironment in Limited-Stage Neuroendocrine-High and -Low Small Cell Lung Cancer. Biology.

[35] Jiang P, Lagenaur CF, Narayanan V (1999). Integrin-associated Protein Is a Ligand for the P84 Neural Adhesion Molecule*. J. Biol. Chem..

[36] Brown EJ, Frazier WA (2001). Integrin-associated protein (CD47) and its ligands. Trends Cell Biol..

[37] Xuan W, Qu Q, Zheng B, Xiong S, Fan G-H (2015). The chemotaxis of M1 and M2 macrophages is regulated by different chemokines. J. Leukoc. Biol..

[38] Zheng Y, Wang Z, Wei S, Liu Z, Chen G (2021). Epigenetic silencing of chemokine CCL2 represses macrophage infiltration to potentiate tumor development in small cell lung cancer. Cancer Lett..

[39] Raulet DH, Gasser S, Gowen BG, Deng W, Jung H (2013). Regulation of ligands for the NKG2D activating receptor. Annu. Rev. Immunol..

[40] Zhao P (2022). c-Myc Targets HDAC3 to Suppress NKG2DL Expression and Innate Immune Response in N-Type SCLC through Histone Deacetylation. Cancers.

[41] Hanahan D, Weinberg RA (2011). Hallmarks of Cancer: The Next Generation. Cell.

[42] Liu Y (2018). NK Cells Mediate Synergistic Antitumor Effects of Combined Inhibition of HDAC6 and BET in a SCLC Preclinical Model. Cancer Res..

[43] Zhu M (2021). Evasion of Innate Immunity Contributes to Small Cell Lung Cancer Progression and Metastasis. Cancer Res..

[44] Saito M (2018). Identification of candidate responders for anti-PD-L1/PD-1 immunotherapy, Rova-T therapy, or EZH2 inhibitory therapy in small-cell lung cancer. Mol. Clin. Oncol..

[45] Gius D (2004). Distinct effects on gene expression of chemical and genetic manipulation of the cancer epigenome revealed by a multimodality approach. Cancer Cell..

[46] Rudin CM (2019). Molecular subtypes of small cell lung cancer: a synthesis of human and mouse model data. Nat. Rev. Cancer..

[47] Gay CM (2021). Patterns of transcription factor programs and immune pathway activation define four major subtypes of SCLC with distinct therapeutic vulnerabilities. Cancer Cell..

[48] Shi Y (2004). Histone Demethylation Mediated by the Nuclear Amine Oxidase Homolog LSD1. Cell.

[49] Shi Y-J (2005). Regulation of LSD1 Histone Demethylase Activity by Its Associated Factors. Mol. Cell..

[50] Augert A (2019). Targeting NOTCH activation in small cell lung cancer through LSD1 inhibition. Sci. Signal..

[51] Bernstein BE (2002). Methylation of histone H3 Lys 4 in coding regions of active genes. Proc. Natl Acad. Sci..

[52] Bernstein BE (2005). Genomic Maps and Comparative Analysis of Histone Modifications in Human and Mouse. Cell.

[53] Santos-Rosa H (2002). Active genes are tri-methylated at K4 of histone H3. Nature.

[54] Pokholok DK (2005). Genome-wide Map of Nucleosome Acetylation and Methylation in Yeast. Cell.

[55] Mohammad HP (2015). A DNA Hypomethylation Signature Predicts Antitumor Activity of LSD1 Inhibitors in SCLC. Cancer Cell..

[56] Takagi S (2017). LSD1 Inhibitor T-3775440 Inhibits SCLC Cell Proliferation by Disrupting LSD1 Interactions with SNAG Domain Proteins INSM1 and GFI1B. Cancer Res..

[57] Oser MG (2019). The KDM5A/RBP2 histone demethylase represses NOTCH signaling to sustain neuroendocrine differentiation and promote small cell lung cancer tumorigenesis. Genes Dev..

[58] Best SA (2020). Harnessing Natural Killer Immunity in Metastatic SCLC. J. Thorac. Oncol..

[59] Mahadevan NR (2021). Intrinsic Immunogenicity of Small Cell Lung Carcinoma Revealed by Its Cellular Plasticity. Cancer Disco..

[60] Nguyen EM (2022). Targeting Lysine-Specific Demethylase 1 Rescues Major Histocompatibility Complex Class I Antigen Presentation and Overcomes Programmed Death-Ligand 1 Blockade Resistance in SCLC. J. Thorac. Oncol..

[61] Shepherd FA (2007). The International Association for the Study of Lung Cancer Lung Cancer Staging Project: Proposals Regarding the Clinical Staging of Small Cell Lung Cancer in the Forthcoming (Seventh) Edition of the Tumor, Node, Metastasis Classification for Lung Cancer. J. Thorac. Oncol..

[62] Torre LA (2015). Global cancer statistics, 2012. Ca. Cancer J. Clin..

[63] Denny SK (2016). Nfib Promotes Metastasis through a Widespread Increase in Chromatin Accessibility. Cell.

[64] Dooley AL (2011). Nuclear factor I/B is an oncogene in small cell lung cancer. Genes Dev..

[65] Wu N (2016). NFIB overexpression cooperates with Rb/p53 deletion to promote small cell lung cancer. Oncotarget.

[66] Semenova EA (2016). Transcription Factor NFIB Is a Driver of Small Cell Lung Cancer Progression in Mice and Marks Metastatic Disease in Patients. Cell Rep..

[67] Fane ME (2017). NFIB Mediates BRN2 Driven Melanoma Cell Migration and Invasion Through Regulation of EZH2 and MITF. eBioMedicine.

[68] Esteller M (2007). Epigenetic gene silencing in cancer: the DNA hypermethylome. Hum. Mol. Genet..

[69] Doi A (2009). Differential methylation of tissue- and cancer-specific CpG island shores distinguishes human induced pluripotent stem cells, embryonic stem cells and fibroblasts. Nat. Genet..

[70] Yang J (2020). Guidelines and definitions for research on epithelial–mesenchymal transition. Nat. Rev. Mol. Cell Biol..

[71] Hou J-M (2012). Clinical Significance and Molecular Characteristics of Circulating Tumor Cells and Circulating Tumor Microemboli in Patients With Small-Cell Lung Cancer. J. Clin. Oncol..

[72] Kim D-W (2016). Genetic requirement for Mycl and efficacy of RNA Pol I inhibition in mouse models of small cell lung cancer. Genes Dev..

[73] Jia D (2018). Crebbp Loss Drives Small Cell Lung Cancer and Increases Sensitivity to HDAC Inhibition. Cancer Disco..

[74] Ko J, Winslow MM, Sage J (2021). Mechanisms of small cell lung cancer metastasis. EMBO Mol. Med..

[75] Vaz M (2017). Chronic Cigarette Smoke-Induced Epigenomic Changes Precede Sensitization of Bronchial Epithelial Cells to Single-Step Transformation by KRAS Mutations. Cancer Cell..

[76] Anzalone G (2019). Cigarette smoke affects the onco-suppressor DAB2IP expression in bronchial epithelial cells of COPD patients. Sci. Rep..

[77] Bersaas A, Arnoldussen YJ, Sjøberg M, Haugen A, Mollerup S (2016). Epithelial-mesenchymal transition and FOXA genes during tobacco smoke carcinogen induced transformation of human bronchial epithelial cells. Toxicol. Vitr..

[78] Liu Y (2015). Epithelial-mesenchymal transition and cancer stem cells, mediated by a long non-coding RNA, HOTAIR, are involved in cell malignant transformation induced by cigarette smoke extract. Toxicol. Appl. Pharmacol..

[79] Bhat SA (2016). Long non-coding RNAs: Mechanism of action and functional utility. Non-Coding RNA Res..

[80] Lu L (2015). Posttranscriptional silencing of the lncRNA MALAT1 by miR-217 inhibits the epithelial–mesenchymal transition via enhancer of zeste homolog 2 in the malignant transformation of HBE cells induced by cigarette smoke extract. Toxicol. Appl. Pharmacol..

[81] Tsai M-C (2010). Long Noncoding RNA as Modular Scaffold of Histone Modification Complexes. Science.

[82] Byers LA (2012). Proteomic Profiling Identifies Dysregulated Pathways in Small Cell Lung Cancer and Novel Therapeutic Targets Including PARP1. Cancer Disco..

[83] Stewart CA (2017). Dynamic variations in epithelial-to-mesenchymal transition (EMT), ATM, and SLFN11 govern response to PARP inhibitors and cisplatin in small cell lung cancer. Oncotarget.

[84] Gardner EE (2017). Chemosensitive Relapse in Small Cell Lung Cancer Proceeds through an EZH2-SLFN11 Axis. Cancer Cell..

[85] Kadoch C, Crabtree GR (2015). Mammalian SWI/SNF chromatin remodeling complexes and cancer: Mechanistic insights gained from human genomics. Sci. Adv..

[86] Fernando TM (2020). Functional characterization of SMARCA4 variants identified by targeted exome-sequencing of 131,668 cancer patients. Nat. Commun..

[87] Concepcion CP (2022). Smarca4 Inactivation Promotes Lineage-Specific Transformation and Early Metastatic Features in the Lung. Cancer Disco..

[88] Yan H-B (2014). Reduced expression of the chromatin remodeling gene ARID1A enhances gastric cancer cell migration and invasion via downregulation of E-cadherin transcription. Carcinogenesis.

[89] Tomihara H (2021). Loss of ARID1A Promotes Epithelial–Mesenchymal Transition and Sensitizes Pancreatic Tumors to Proteotoxic Stress. Cancer Res..

[90] Sasaki T (2022). Tumor progression by epithelial-mesenchymal transition in ARID1A- and SMARCA4-aberrant solid-type poorly differentiated gastric adenocarcinoma. Virchows Arch..

[91] Kim J (2021). SMARCA4 Depletion Induces Cisplatin Resistance by Activating YAP1-Mediated Epithelial-to-Mesenchymal Transition in Triple-Negative Breast Cancer. Cancers.

[92] Lamar JM (2012). The Hippo pathway target, YAP, promotes metastasis through its TEAD-interaction domain. Proc. Natl Acad. Sci..

[93] Ireland AS (2020). MYC Drives Temporal Evolution of Small Cell Lung Cancer Subtypes by Reprogramming Neuroendocrine Fate. Cancer Cell..

[94] Terashima M, Ishimura A, Wanna-udom S, Suzuki T (2017). Epigenetic regulation of epithelial-mesenchymal transition by KDM6A histone demethylase in lung cancer cells. Biochem. Biophys. Res. Commun..

[95] Zha L (2016). Epigenetic regulation of E-cadherin expression by the histone demethylase UTX in colon cancer cells. Med. Oncol..

[96] Choi H-J (2015). UTX inhibits EMT-induced breast CSC properties by epigenetic repression of EMT genes in cooperation with LSD1 and HDAC1. EMBO Rep..

[97] Taki M (2021). Tumor Immune Microenvironment during Epithelial–Mesenchymal Transition. Clin. Cancer Res..

[98] Mullins RDZ, Pal A, Barrett TF, Heft Neal ME, Puram SV (2022). Epithelial–Mesenchymal Plasticity in Tumor Immune Evasion. Cancer Res..

[99] Qiu Z (2019). CDYL promotes the chemoresistance of small cell lung cancer by regulating H3K27 trimethylation at the CDKN1C promoter. Theranostics.

[100] Yang F (2021). LncRNA HOTAIR regulates the expression of E-cadherin to affect nasopharyngeal carcinoma progression by recruiting histone methylase EZH2 to mediate H3K27 trimethylation. Genomics.

[101] Fillmore CM (2015). EZH2 inhibition sensitizes BRG1 and EGFR mutant lung tumours to TopoII inhibitors. Nature.

